# RETRACTION: Impact of Enhanced Recovery After Surgery Protocols on Surgical Site Wound Infection Rates in Urological Procedures

**DOI:** 10.1111/iwj.70610

**Published:** 2025-04-21

## Abstract

**RETRACTION:** CaiY. , CaiX. , ZhangX. , ZhuJ. , and ChenW. , “Impact of Enhanced Recovery After Surgery Protocols on Surgical Site Wound Infection Rates in Urological Procedures,” International Wound Journal 21, no. 1 (2024): e14582, 10.1111/iwj.14582.38272818 PMC10789582

The above article, published online on 15 January 2024, in Wiley Online Library (http://onlinelibrary.wiley.com/), has been retracted by agreement between the journal Editor in Chief, Professor Keith Harding; and John Wiley & Sons Ltd. Following an investigation by the publisher, all parties have concluded that this article was accepted solely on the basis of a compromised peer review process. The editors have therefore decided to retract the article. The authors did not respond to our notice regarding the retraction.

**RETRACTION:** Y. Cai , X. Cai , X. Zhang , J. Zhu , and W. Chen , “Impact of Enhanced Recovery After Surgery Protocols on Surgical Site Wound Infection Rates in Urological Procedures,” International Wound Journal 21, no. 1 (2024): e14582, 10.1111/iwj.14582.38272818 PMC10789582

The above article, published online on 15 January 2024, in Wiley Online Library (http://onlinelibrary.wiley.com/), has been retracted by agreement between the journal Editor in Chief, Professor Keith Harding; and John Wiley & Sons Ltd. Following an investigation by the publisher, all parties have concluded that this article was accepted solely on the basis of a compromised peer review process. The editors have therefore decided to retract the article. The authors did not respond to our notice regarding the retraction.

